# Exploring neurokinin-1 receptor antagonism for depression with structurally differentiated inhibitors

**DOI:** 10.1038/s12276-025-01576-0

**Published:** 2025-11-28

**Authors:** Hyeijung Yoo, Kyung-Jun Boo, Lan Phuong Nguyen, Jong-Ik Hwang, Cheol Soon Lee, Soo Hyun Yang, Se Jin Jeon, Hong-Rae Kim, Hyun Kim

**Affiliations:** 1 Department of Anatomy, College of Medicine, Korea University, Seoul, Republic of Korea; 2 Department of Pharmacology, College of Medicine and Center for Neuroscience Research, IBST, Konkuk University, Seoul, Republic of Korea; 3 Department of Biomedical Sciences, College of Medicine, Korea University, Seoul, Republic of Korea; 4 Department of Pharmacology, College of Medicine, Hallym University, Chuncheon, Republic of Korea

**Keywords:** Drug development, Depression

## Abstract

The neurokinin-1 receptor (NK1R) has been investigated as a potential target for major depressive disorder owing to its role in stress regulation and neuroinflammation. However, clinical trials of NK1R antagonists have yielded inconsistent results, leaving it unclear whether these outcomes reflect limitations of NK1R as a therapeutic target or shortcomings inherent to the clinical candidates tested. The majority of previously developed NK1R antagonists contain a 3,5-bis-trifluoromethylphenyl moiety, which enhances receptor binding but may also influence drug metabolism, pharmacokinetics or receptor interactions, potentially affecting therapeutic efficacy. Whether structurally distinct NK1R antagonists exhibit different antidepressant potential remains an open question. Here we used computational approaches to identify NK1R antagonists lacking the 3,5-bis-trifluoromethylphenyl group and evaluated their effects in preclinical models of depression. Several compounds exhibited NK1R antagonistic activity and reduced depressive-like behaviors, with compound #15 demonstrating the most pronounced effects. Molecular docking and molecular dynamics simulations revealed a distinct binding mode for compound #15, characterized by a hydrogen bond interaction with Asn109 and π–π stacking with His197, suggesting structural differences that may influence NK1R modulation. These findings support the potential of structurally diverse NK1R antagonists to modulate behavior and neuroinflammatory responses in preclinical models. While the relevance of these structural differences to clinical outcomes remains to be determined, our results provide a preliminary framework for further investigation of chemically novel NK1R antagonists in the context of major depressive disorder.

## Novel NK1R antagonists show promise against depression

Major depressive disorder is a common mental health issue affecting millions worldwide. This study investigates a renewed approach targeting the neurokinin-1 receptor that is linked to stress and inflammation in the brain. Researchers used computer models to find new NK1R antagonists without a specific chemical group that might limit effectiveness. They tested these compounds in lab settings and on mice to see if they could reduce depression-like symptoms and brain inflammation. One compound, called #15, showed promise by reducing inflammation and depressive behaviors in mice. This suggests it could be a potential treatment for MDD. The study highlights the importance of exploring different chemical structures for developing better antidepressants. Future research will focus on understanding how these new compounds work in the body and their potential as treatments for depression, especially in cases linked to inflammation.

This summary was initially drafted using artificial intelligence, then revised and fact-checked by the author.

## Introduction

Major depressive disorder (MDD) is a leading cause of disability worldwide, affecting over 300 million individuals and imposing a substantial socioeconomic burden^1^. Although multiple pharmacological treatments are available, their efficacy varies substantially among patients, reflecting the heterogeneous nature of MDD pathophysiology^2–4^. These limitations highlight the need for alternative therapeutic strategies beyond currently available antidepressants^5^.

The neurokinin-1 receptor (NK1R) has been investigated as a potential target for MDD owing to its role in stress regulation and neuroinflammation^6^. Substance P (SP), the endogenous ligand for NK1R, is elevated in the cerebrospinal fluid of patients with MDD and has been implicated in neuroinflammatory processes increasingly associated with depressive pathology^7–10^. Preclinical studies in rodents and nonhuman primates have demonstrated that NK1R antagonists produce antidepressant-like effects, supporting their potential therapeutic relevance^11,12^.

However, despite promising preclinical findings, NK1R antagonists, including Aprepitant, failed to show significant efficacy in phase III clinical trials, leading to the discontinuation of their development for depression^12–14^. Although trial design and methodological factors may have contributed to these outcomes, it remains unclear whether the pharmacological properties of existing NK1R antagonists also played a role in their limited success^15^.

A structural feature common to many previously developed NK1R antagonists is the 3,5-bis-trifluoromethylphenyl (TFMP) group (Supplementary Fig. 1). While this motif is known to enhance receptor binding affinity, it may also influence drug metabolism, pharmacokinetics or receptor interactions, thereby influencing therapeutic efficacy^16–18^. Despite this, no systematic studies have investigated whether modifying or removing TFMP could improve the antidepressant potential of NK1R antagonists. Although the relationship between compound structure and clinical efficacy remains to be established, evaluating structurally distinct NK1R antagonists offers an initial step toward exploring how chemical modifications may influence biological outcomes. To address this, we utilized computational approaches to identify NK1R antagonists with alternative structural features, evaluated their antidepressant-like effects in preclinical models of depression using behavioral assessments and analyzed markers of neuroinflammation at the mRNA level. Our findings suggest that the structurally distinct antagonists identified in this study exhibit antidepressant-like effects, providing renewed evidence for further exploration of NK1R antagonism as a therapeutic strategy for MDD.

## Materials and methods

### Dataset

#### Training dataset

A dataset containing ChEMBL ID and activity measurements, including half-maximal inhibitory concentration (IC₅₀), half-maximal effective concentration (EC₅₀), inhibition constant (Kᵢ), and dissociation constant (K_d_) values, was obtained from the ChEMBL database (https://www.ebi.ac.uk/chembl/, 5,997 results) and NCBI PHAROS database (https://pharos.nih.gov, 1,632 results). After removing duplicates, 2,499 ligands were included in the study. The pChEMBL value was calculated as −log_10_(effective value) for prediction. Dimensionality reduction of ligand structures was conducted by *t*-distributed stochastic neighbor embedding with the Rtsne package (v 0.16) in R 4.2.2.

#### Screening dataset

The Enamine Screening Collection, which contains 2,681,264 molecules, was obtained from https://enamine.net/compound-collections/screening-collection and used for virtual screening.

#### Virtual screening

##### Quantitative structure–activity relationship (QSAR) model generation

QSAR models were developed to predict the pChEMBL value using KNIME (version 4.4.4) with the Schrödinger extension (version 21.4.135) and DeepChem in Maestro 12.8 (Schrödinger 2021-2 version). Four different approaches were used: random forest, simple regression, AutoQSAR and DeepChem. The dataset was divided into a training set (70%, 1,749 ligands) and a test set (30%, 750 ligands). For AutoQSAR and DeepChem, the SMILES (Simplified Molecular-Input Line-Entry System) string for each ligand was converted to .Mae file format. For other methods, the SMILES strings were transformed into Morgan fingerprints (radius, 2; number of bits, 1,024) using RDkit nodes in KNIME. Predictions were evaluated using the coefficient of determination (*R*^2^) and root mean square error/deviation (RMSE/RMSD).

$$R^{2} = 1-\frac{\mathrm{SSR}}{\mathrm{SST}} = 1-\frac{\sum_{i=1}^{n} (y_i - \hat{y}_i)^{2}}{\sum_{i=1}^{n} (y_i - \bar{y})^{2}},\qquad {\text{RMSE}}\,({\text{RMSD}})=\sqrt{\frac{1}{n} \sum_{i=1}^{n} (y_i - \hat{y}_i)^{2}}$$

##### Ligand clustering

Ligands were clustered based on Murcko scaffolds and ECFP4 fingerprints using Tanimoto coefficients, and hierarchical clustering was performed with a distance threshold of 0.75 to form 35 clusters. Fifteen ligands were manually selected for in vitro experiments considering cluster assignment, prediction score and core structure.

##### Similarity search

Similar ligands to compound #1 were searched using infiniSee (BioSolveIT) in the Enamine REAL space containing 3.4 × 10^10^ ligands and the KnowledgeSpace with 2.9 × 10^14^ ligands. The predicted pChEMBL value of the top 200,000 similar ligands was calculated with the generated DeepChem model. Similar ligands were filtered with the predicted pChEMBL score (>9.5). Among 40 ligands, 8 ligands were selected on the basis of core structure.

### Molecular docking and MD simulation

Molecular docking experiments were performed using Schrödinger Glide software (version 2022-4), and molecular dynamics (MD) simulations were conducted using Desmond on the Schrödinger platform. All calculations were executed in a Linux environment.

#### Preparation of protein and ligands

The crystal structure of the protein (PDB ID: 6HLO) was imported into Maestro and prepared for docking using the Protein Preparation Workflow with the OPLS4 force field. The receptor grid for docking was defined based on the position of the bound ligand, Aprepitant, without further modification. The compounds were prepared for docking using the LigPrep function, which generated all possible stereoisomers and states at a target pH of 7.0 ± 2.0.

#### Molecular docking

The prepared compounds were docked to the protein structure using the Glide software with extra precision (XP) settings. This docking process generated protein–ligand complexes for further analysis and exploration.

#### MD simulation

The protein–ligand complexes obtained from the molecular docking were utilized to build the system for MD simulations. The simulation setup included the placement of a POPC (300 K) membrane model and the addition of counter ions to neutralize the system, with TIP3P as the solvent system. The MD simulation was performed for a duration of 500 ns using the ensemble class NPγT at a temperature of 300.0 K. The simulation trajectory was subsequently analyzed using the Simulation Interactive Diagram.

### In vitro screening

The FLIPR calcium 6 assay (Molecular Devices) was used for evaluating IC_50_ and the dose–response curve of each ligand. hTACR1-HEK293 cells were seeded in 96-well black clear-bottom plates at a cell density of 3 × 10^4^ cells per well. The following day, the cells were loaded with the FLIPR calcium 6 dye mixed at 1:1 to medium with 2.5 mM probenecid. The assay plate was wrapped with aluminum foil and incubated for 2 h at 37 °C in a humidified CO_2_ incubator. Each concentration of chemicals was dissolved in 1× Hank’s Balanced Salt Solution (plus 20 mM HEPES buffer, pH 7.4) and added to the well. The plate was incubated for 30 min at 37 °C before measuring. The final concentration of dimethyl sulfoxide was 0.5% in each well. Then, 1 nM SP-induced intracellular calcium mobilization was examined by monitoring the fluorescence intensity (excitation at 485 nm, emission at 525 nm, cutoff filter set at 515 nm) using a Flex Station 3 multimode microplate reader. Changes in fluorescence signal after SP addition were normalized to its starting signal and denoted as *F*/*F*_0_.

### Animal models of depression

#### Animals

All experimental procedures with animals were approved by Korea University and Konkuk University Institution of Animal Care and Use Committee (study approval numbers KOREA-2021-0202 and KU22186) and were performed according to university and the ARRIVE (Animal Research: Reporting of In Vivo Experiments) guidelines. Seven-week-old male C57BL/6 mice (Orient Bio) were group housed under a 12-h light/dark cycle and given ad libitum access to food and water. After arrival, animals were allowed 1 week to habituate to the facility before experiments and randomly divided into experimental and control groups.

#### Ligand preparation

The ligands were dissolved at a dose of 10 mg/kg in the vehicle (10% dimethyl sulfoxide and 10% Kolliphor in 0.9% saline) and intraperitoneally injected into each animal in experimental groups.

#### LPS model

To generate the lipopolysaccharide (LPS) model, 0.25 mg/kg of LPS from *E. coli* O26:B6 (Sigma, L3755) was dissolved in 0.9% saline and injected intraperitoneally. The control group was treated with 0.9% saline instead of LPS injection. The mice received injections of either vehicle, compound #1, compound #15 or Aprepitant (Tocris Bioscience) on day 1, 1 h before LPS injection and on day 2.

#### RS model

To induce depressive-like behaviors in mice, restraint stress (RS) was applied using 50-ml conical tubes with many holes for 20 days, 2 h per day. From day 11 to 20, mice received injections of either vehicle or compound #15.

#### SI stress model

To generate the social isolation (SI) stress model, the animals were single-caged for 14 days. From day 6 to 13, mice received injections of either vehicle or compound #15.

### qRT–PCR

Four hours after LPS injection, the frontal lobe and hippocampus were isolated from the animal brain and placed in TRIzol solution (Ambion). Total RNA sample (2 μg) was reverse-transcribed into cDNA using Moloney Murine Leukemia Virus reverse transcriptase (M-MLV RT; Promega) and oligo (dT) primer (Novagen). Quantitative reverse transcription polymerase chain reaction (qRT–PCR), which quantifies gene expression levels, was performed using 0.5 μg of the reverse-transcription generated cDNA and specific primer sets (Supplementary Table 3). PCR amplification with iQ SYBR Green Supermix was performed in triplicate using the CFX96 Touch-Time System (Bio-Rad). The final products of qRT–PCR were electrophoresed on 2% agarose gels and visualized with SafeView Nucleic Acid Stain (G108, Applied Biological Materials). Cycle threshold (Ct) values at which the fluorescent signal exceeded the background were determined by qRT–PCR, and expression values for each gene were normalized to expression values of GAPDH. Relative quantification to calculate fold change was performed using the comparative Ct method (ΔΔCt).

### Behavior experiments

#### TST

On day 4 after LPS administration, the tail suspension test (TST) was conducted. A four-chamber apparatus divided by nontransparent acrylic partitions was used, and the mice were suspended in each chamber by the tail. A video was recorded for 6 min, and the last 4 min were manually scored for immobility time.

#### FST

On day 21 in the RS model and day 12 in the SI model, the forced swim test (FST) was performed. During each trial, mice were placed in a test cylinder (diameter 15 cm, height 25 cm) filled with water regulated at 25 ± 1 °C, and a video was recorded for 6 min. Immobility time was manually scored during the last 4 min of each trial.

#### OFT

On day 24 in the RS model and day 13 in the SI model, the open field test (OFT) was conducted to measure the general locomotor activity of mice. Each mouse was individually placed in an open box (40 × 40 × 40 cm) for 10 min. The locomotion of mice was tracked using EthoVision XT 11.5 (Noldus). The total distance moved and movement duration were measured for analysis.

### Statistical analysis

Statistical analysis was conducted with IBM SPSS Statistics 24 for Windows software (IBM). Comparisons between groups were analyzed using one-way analysis of variance (ANOVA), followed by Tukey’s honestly significant difference (HSD) as a post-hoc test. The harmonic mean of the group sizes was used for unequal-sized groups. *P* < 0.05 was considered significant, and values were expressed as mean ± s.e.m.

## Results

### Machine learning-powered virtual screening identifies structurally distinct NK1R antagonists

**Fig. 1:**
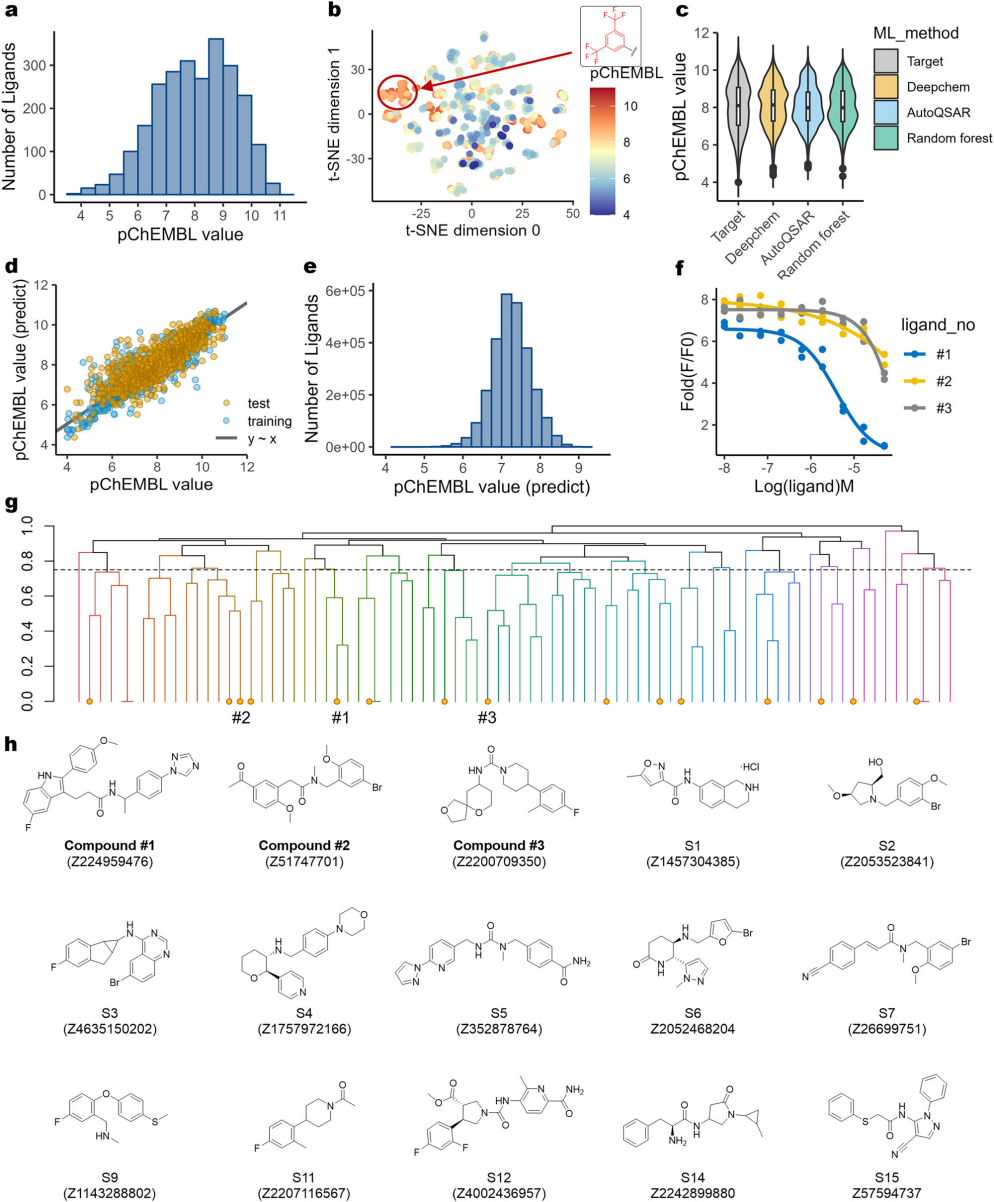
Machine learning-powered virtual screening identifies antagonists devoid of the TFMP group. **a** The number of ligands distributed across pChEMBL values ranging from 4 to 11, used in developing the screening models. **b** Two-dimensional visualization of the dataset based on their structural features, with the circled portion representing structures containing the TFMP group. **c** The distribution of predicted pChEMBL values using different machine learning algorithms. **d** Correlation between predicted and experimental pChEMBL values using the DeepChem model. **e** The distribution of predicted pChEMBL values from screening the Enamine Screening Collection. **f** Dose–response curve of the three active compounds determined by the FLIPR calcium 6 assay. **g** Clustering based on structural similarity, where circles indicate compounds selected for in vitro validation. **h** Structures of compounds selected for in vitro validation.

To identify novel chemical scaffolds of NK1R antagonists devoid of the TFMP group, we developed a machine learning-based predictive model for ligand activity. A dataset of 2,499 NK1R ligands with experimentally determined pChEMBL values (range 4–11) was compiled for model training (Fig. 1a). The chemical diversity of these ligands was visualized using *t*-distributed stochastic neighbor embedding, which mapped their distribution in a bidimensional chemical space, revealing distinct structural clusters (Fig. 1b). Notably, compounds containing the TFMP motif formed a separate cluster, indicating that the model effectively captured structural relationships among known NK1R antagonists.

To assess predictive performance, we evaluated multiple computational models, including random forest, simple regression, AutoQSAR and DeepChem, based on their *R*² and RMSD values (Fig. 1c and Supplementary Table 1). Among these, DeepChem demonstrated the highest predictive accuracy, achieving *R*² = 0.873, RMSD = 0.481 in the training set and R² = 0.674, RMSD = 0.771 in the test set (Fig. 1d and Supplementary Table 1). Given its performance, we used the DeepChem model to screen the Enamine Screening Collection, a commercially available compound library (Fig. 1e).

From this screening, 83 compounds with predicted pChEMBL values >9.0 were selected for further evaluation. Hierarchical clustering was applied to group structurally similar compounds, followed by a drug-likeness assessment using Lipinski’s Rule of Five (Fig. 1g). This filtering process prioritized 15 candidate compounds for in vitro validation (Fig. 1h).

To assess their NK1R antagonistic activity, we performed a FLIPR calcium 6 assay. Among the tested compounds, compound #1, featuring an aryl indole core, exhibited the highest activity, with a single-digit micromolar IC_50_, while two additional compounds (#2 and #3) demonstrated weaker inhibitory effects (Fig. 1f). These findings suggest that our machine learning-powered screening approach identified NK1R antagonists with structurally distinct scaffolds compared with previously investigated compounds.

### Compound #1 reduces neuroinflammation and alleviates depressive-like behavior in an LPS-induced model

**Fig. 2:**
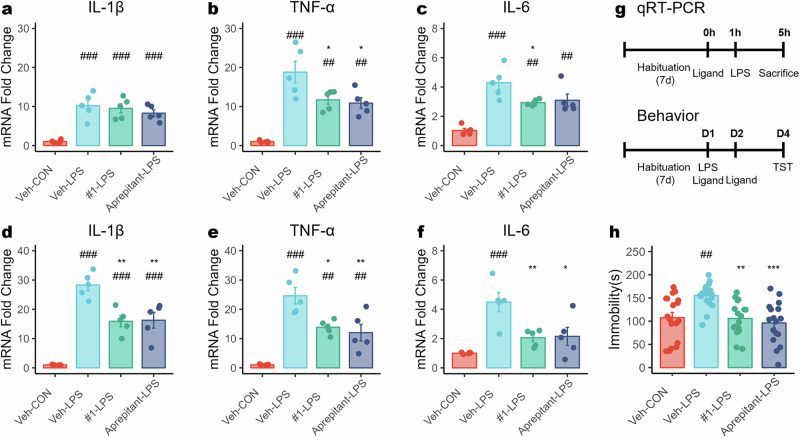
NK1R antagonists reduce neuroinflammation and despair-like behavior in LPS model. **a**–**f** Expression levels of proinflammatory cytokines IL-1β (**a** and **d**), TNF-α (**b** and **e**) and IL-6 (**c** and **f**) measured in the frontal cortex (**a**–**c**) and in the hippocampus (**d**–**f**) with or without compound pretreatment. The following groups were included: CON (*n* = 5), LPS (*n* = 5), compound #1 + LPS (*n* = 5) Aprepirant + LPS (*n* = 5). **g** The experimental paradigm for qRT–PCR for inflammatory cytokines, and behavioral testing on the LPS model. **h** TST with or without compound pretreatment. The following groups were included: CON (*n* = 18), LPS (*n* = 18), LPS + compound #1 (*n* = 17) LPS + A (Aprepitant, *n* = 17). Statistical significance was measured with ANOVA (post-hoc Tukey HSD) and denoted as #*P* < 0.05, ##*P* < 0.01, ###*P* < 0.001, indicating statistical significance compared with the Veh-CON group, and *P < 0.05, **P < 0.01, ***P < 0.001, compared with the Veh-LPS group.

To evaluate potential anti-inflammatory effects in the central nervous system, we measured the mRNA expression of IL-1β, TNF-α and IL-6 in the frontal cortex and hippocampus of LPS-treated mice following administration of vehicle, compound #1 (10 mg/kg), or Aprepitant (10 mg/kg; Fig. 2g). The LPS-treated group exhibited significantly elevated mRNA levels of IL-1β, TNF-α and IL-6 compared with the control group, confirming the inflammatory response induced by LPS. However, treatment with compound #1 (#1-LPS) or Aprepitant (Aprepitant-LPS) significantly reduced TNF-α and IL-6 levels in the frontal cortex (Fig. 2a–c) and IL-1β, TNF-α and IL-6 levels in the hippocampus (Fig. 2d–f) compared with the LPS-treated group. Both compound #1 and Aprepitant reduced these elevations (Fig. 2a–f), showing comparable effects in this assay. These findings suggest that NK1R antagonism attenuates LPS-induced neuroinflammatory responses in the central nervous system.

Next, we examined whether compound #1 could mitigate depressive-like behavior using the TST (Fig. 2g). The LPS-treated group exhibited a significant increase in immobility time, indicative of depression-like behavior, compared with the control group. However, mice pretreated with compound #1 or Aprepitant demonstrated a significant reduction in immobility time compared with the LPS-treated group (Fig. 2h). These results suggest that NK1R antagonism alleviates neuroinflammation-induced depressive-like symptoms, supporting the potential antidepressant effects of NK1R antagonists.

### Identification of compound #15 as a more potent NK1R antagonist

Encouraged by the activity of compound #1, we sought to identify a more potent NK1R antagonist for further investigation. To achieve this, we used infiniSee to explore structural analogs of compound #1 across a larger chemical space. Using a similarity-based approach, we predicted pChEMBL values for the top 200,000 compounds and selected 40 candidates with predicted values exceeding 9.5. From these, compounds were clustered based on structural similarity, and eight representative analogs were selected along with four structurally similar ligands from the Enamine Screening Collection for in vitro validation (Supplementary Fig. 2).

**Fig. 3:**
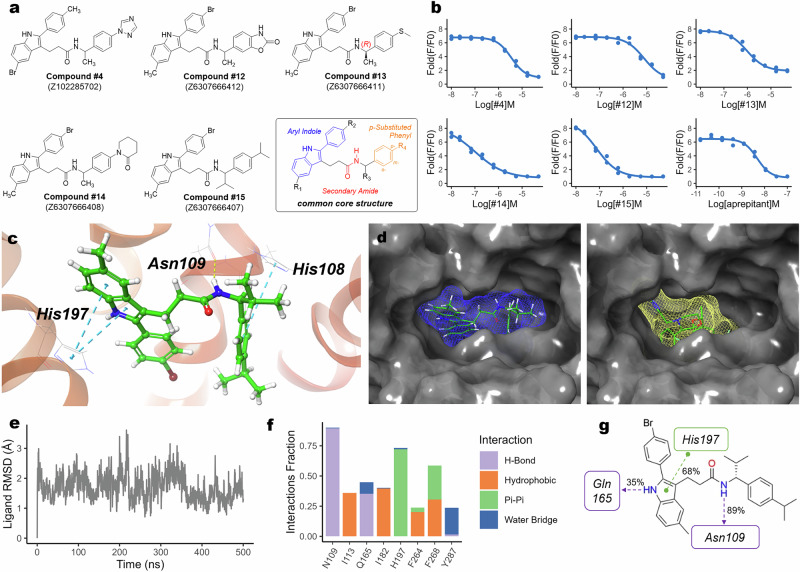
NK1R antagonistic activity of aryl indole analogs and binding mode analysis of representative compound #15. **a** Structures of compounds exhibiting stronger antagonistic activity than compound #1, derived from the analog search, along with their common structural motif. **b** Dose–response curves for the five most potent compounds, determined using a calcium 6 assay. **c** The binding pose of compound #15 as determined by molecular docking studies (PDB ID: 6HLO). π–π interactions are depicted as dashed blue lines, and hydrogen bonding interactions are shown in yellow. **d** A surface map comparison of compound #15 (blue) and Aprepitant (yellow). **e**–**g** Analysis of compound #15 based on a 500-ns MD simulation: deviation of the ligand from its initial position represented as a RMSD plot (**e**); key interacting residues (**f**); and interacting components of compound #15 (**g**). Hydrogen bonding interactions are depicted as purple dashes and π–π interaction in green. Percentages represent interaction frequencies.

All selected compounds shared a core aryl indole scaffold linked to a secondary amide, with structural variations at the R_1_–R_4_ positions (Fig. 3a). This common motif appeared to contribute to high predicted pChEMBL values, consistent with previous reports identifying aryl indoles as NK1R inhibitors^19^. Further analysis suggested that small substituents at R_1_ and R_2_ had minimal impact on antagonistic activity, a trend supported by compounds #1 and #4, as well as prior literature^20^. However, despite overall structural similarity, activity was predominantly influenced by modifications at the R_4_ position.

Notably, *para*-substituted phenyl rings at R_4_ were associated with higher inhibitory activity, whereas replacing the phenyl ring with a nonaromatic moiety (compounds #5 and #6) or pyridine (compounds #7 and #8) resulted in a complete loss of activity. Similarly, *meta*-substitutions at R_4_ abolished activity, suggesting that specific electronic and steric properties are important for maintaining NK1R antagonism. A brief structure–activity relationship (SAR) analysis (Supplementary Table 2) supported these observations. Importantly, none of these compounds contains the TFMP group common in many legacy NK1R antagonists.

Among the validated compounds, five exhibited stronger inhibitory activity than compound #1 (Fig. 3a, b), with compound #15 emerging as the most potent, exhibiting an IC_50_ of 79.5 nM. Based on this potency, compound #15 was selected for further study.

### Molecular docking and MD simulation reveal a unique binding mode of compound #15 in NK1R

To gain insight into how the antagonists interact with NK1R, we conducted molecular docking and MD simulations to identify key protein–ligand interactions. Docking Aprepitant to NK1R (PDB: 6HLO), followed by a 500-ns MD simulation, revealed that hydrogen bonding with Gln165 was the primary stabilizing interaction between the ligand and receptor (Supplementary Fig. 3). This computational observation aligned with previously reported protein–ligand interactions from a recently solved crystal structure^21^, supporting the validity of our predictions.

Molecular docking of compound #15 with NK1R (PDB: 6HLO) revealed a binding conformation distinct from Aprepitant, characterized by two parallel planes linked by an alkyl linker (Fig. 3c). Surface map analysis showed that compound #15 occupies a broader region of the binding pocket compared with Aprepitant (Fig. 3d), suggesting that its structural features may lead to differences in receptor interactions^22^.

A 500-ns MD simulation of compound #15 demonstrated low RMSD values, with an average displacement of 1.706 Å from the originally docked position, indicating that the ligand remained stable throughout the simulation (Fig. 3e and Supplementary Fig. 4). Further analysis identified a hydrogen bond interaction with Asn109, along with a π–π stacking interaction with His197 (Fig. 3f). The hydrogen bond with Asn109 specifically involved the amide group of compound #15, a structural motif commonly identified during virtual screening (Fig. 3g).

While aryl indole scaffolds have been previously reported as NK1R inhibitors, prior compounds primarily featured tertiary amides, which lack the hydrogen necessary for hydrogen bonding at this position^19,20^. The unique binding mode of compound #15, particularly its novel hydrogen bonding and expanded binding pocket interactions, highlights a previously unrecognized structural feature among NK1R antagonists.

### Compound #15 alleviates depressive-like behavior across multiple mouse models

**Fig. 4:**
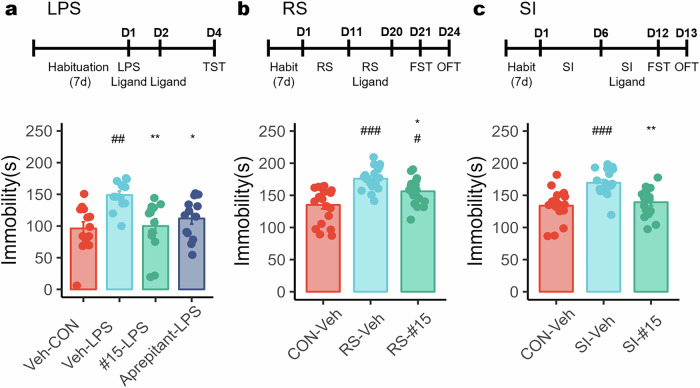
Compound #15 reduces despair-like behavior in animal models of depression. Experimental paradigm for LPS model generation and behavioral testing and measurement of despair-like symptoms with or without compound #15 pretreatment. **a** TST on LPS model; The following groups were included: CON (*n* = 13), LPS (n = 13), LPS + compound #15 (*n* = 13) LPS + A (Aprepitant, *n* = 13). **b**, **c** FST on RS (**b**) and SI (**c**) model; The following groups were included: CON (*n* = 19), RS (*n* = 19), RS + compound #15 (*n* = 19) in **b**, and CON (*n* = 17), SI (*n* = 17), SI + compound #15 (*n* = 16) in **c**. Statistical significance was measured with ANOVA (post-hoc Tukey HSD) and denoted as #*P* < 0.05, ##*P* < 0.01, ###*P* < 0.001, indicating statistical significance compared with the Veh-CON group, and *P < 0.05, **P < 0.01, ***P < 0.001, compared with the Veh-LPS group.

To further evaluate the antidepressant-like effects of compound #15, we assessed its activity in multiple mouse models of depression. First, we examined whether compound #15 could replicate the effects of compound #1 and Aprepitant in the LPS-induced depression model. In the TST, treatment with compound #15 significantly reduced immobility time compared with the LPS-treated group, suggesting a correlation between in vitro NK1R antagonism and behavioral improvements (Fig. 4a).

Next, we investigated the effects of compound #15 in RS and SI models. In the FST, both RS-induced and SI-induced groups exhibited a significant increase in immobility time, indicative of despair-like behavior, compared with the control group. Locomotion analysis in the OFT showed no significant changes in RS groups, whereas the SI-induced group displayed increased locomotion (Supplementary Fig. 5).

In both models, treatment with compound #15 led to a notable reduction in immobility time, suggesting a potential attenuation of stress-induced depressive behavior (Fig. 4b, c). These findings provide additional support for the therapeutic relevance of NK1R antagonism in depression models.

## Discussion

The role of SP and its receptor NK1R in modulating emotional behaviors has been extensively studied, with evidence suggesting its involvement in anxiety and depressive symptoms^11,12^. Mice lacking NK1R or TAC1 (which encodes SP) exhibit altered anxiety- and despair-like behaviors^23,24^, and intracerebroventricular administration of an NK1R antagonist has been shown to modulate anxiety-like behavior in the elevated plus maze^25^. In addition, stress-induced increases in SP levels in the amygdala have been associated with heightened anxiety-like behavior, and NK1R antagonism in this region was reported to attenuate these effects in rats^26^. The behavioral effects of compound #1 and compound #15 in this study align with these findings, as both compounds reduced depressive-like behaviors across multiple mouse models of depression.

Beyond its role in emotional regulation, SP–NK1R signaling has been implicated in neuroinflammation, a process increasingly recognized as relevant to MDD. Inflammatory mediators can alter neurotransmitter signaling, synaptic plasticity and neuronal function, all of which have been linked to depressive pathology^10,27,28^. For example, proinflammatory cytokines can increase glutamate release while inhibiting glutamate reuptake, leading to excitotoxicity^29^. Moreover, neuroinflammation has been shown to increase serotonin reuptake and reduce dopamine production, further exacerbating mood disturbances^30,31^. SP and NK1R are expressed in immune cells and contribute to neurogenic inflammation, including leukocyte infiltration, microglial activation and cytokine release^8,32^. Prior studies suggest that NK1R inhibition or SP deletion mitigates neuroinflammatory responses, including oxidative stress and proinflammatory cytokine production^33^.

Consistent with these findings, this study demonstrates that compound #1 reduced levels of proinflammatory cytokines. The aryl indole analogs identified in this work exhibit NK1R antagonistic activity and, as indicated by the effects of compound #1, also show anti-neuroinflammatory properties. These effects were particularly notable in the frontal cortex and hippocampus, regions implicated in mood regulation and stress processing. The frontal cortex, a region susceptible to chronic stress, projects to multiple brain areas responsible for emotion and reward regulation^34^. In rodents, NK1R inhibition in the prefrontal cortex has been shown to modulate stress-induced neurotransmitter release, supporting its role as a target for mood disorders^24,31,35,36^. Likewise, NK1R antagonism in the hippocampus has been linked to modulation of microglial activity and cytokine expression, suggesting a role in stress-related neuroinflammation^37,38^. Given that a subset of patients with MDD exhibit elevated neuroinflammation^39,40^, NK1R antagonists with anti-inflammatory properties could be therapeutic candidates, particularly in patients with concurrent inflammatory conditions. However, additional research is needed to assess whether NK1R antagonists are particularly effective in inflammation-associated depression subtypes or subgroups with heightened neuroinflammatory profiles, which in turn could guide biomarker-driven clinical trials.

The integration of in silico methodologies has significantly improved early drug discovery by facilitating efficient screening and molecular interaction predictions^41^. In this study, machine learning-assisted virtual screening enabled the identification of NK1R antagonists devoid of the TFMP group, followed by molecular docking and MD simulations to predict their binding interactions. This approach led to the identification of compound #1, which exhibited antidepressant-like effects and neuroinflammatory modulation in the LPS model. Further refinement resulted in compound #15, a more potent NK1R antagonist, which demonstrated behavioral efficacy in multiple preclinical models, including LPS-, RS- and SI-induced models. While these findings provide supporting evidence for NK1R antagonism in depression, further studies are required to determine whether these structurally novel antagonists offer advantages over previously tested compounds.

Early NK1R antagonists demonstrated strong preclinical antidepressant effects but failed to show consistent efficacy in phase III clinical trials. However, clinical failure does not necessarily indicate target invalidity, as multiple confounding factors beyond SP–NK1R signaling may influence therapeutic outcomes. For instance, compound-specific pharmacological properties, complex neurobiological mechanisms underlying mood disorders, limitations in trial design and heterogeneous patient populations could all have contributed to inconsistent results. Notably, the shared TFMP core among these early antagonists raises the possibility that their pharmacokinetic or pharmacodynamic properties contributed to their lack of clinical success. While our study does not directly prove that structural differences account for the failure of earlier compounds, it demonstrates that NK1R antagonists with alternative chemical scaffolds can elicit antidepressant-like effects and reduce neuroinflammation in preclinical models. These findings support the pursuit of structurally distinct NK1R antagonists as part of a broader effort to reassess this target in depression, especially in subtypes with inflammatory involvement. Thus, our work is an initial step toward expanding the chemical diversity of NK1R-targeting compounds, providing a foundation for reevaluating NK1R as a viable antidepressant target.

Despite promising preclinical observations, successful translation to clinical application will require a comprehensive evaluation of pharmacokinetics, receptor occupancy and potential off-target effects. Although in silico predictions suggest that compound #15 exhibits distinct pharmacological properties compared with Aprepitant (Supplementary Table 4), these remain theoretical and require empirical validation. Future studies should focus on optimizing drug metabolism, receptor selectivity and in vivo efficacy to better assess the clinical viability of these compounds. A systematic approach integrating SAR studies, metabolic stability assessment and receptor engagement profiling will be necessary to determine the true therapeutic potential of these newly identified NK1R antagonists. Taken together, our results provide a foundation for optimizing NK1R antagonists and underscore the importance of structural diversity, which could lead to new therapeutic options in treatment-resistant or inflammation-associated depression.

## Supplementary information

Supplementary Information
