# Supplementary material for: Exploring neurokinin-1 receptor antagonism for depression with structurally differentiated inhibitors
Source: Exp Mol Med. 2025 Nov 28;57(11):2699–706. doi: 10.1038/s12276-025-01576-0 (PMC12686497; doi:10.1038/s12276-025-01576-0)
Supplement: Supplementary file 1 — Supplementary Information [file 12276_2025_1576_MOESM1_ESM.pdf]

SUPPLEMENTARY INFORMATION

Title: Exploring Neurokinin-1 Receptor Antagonism for Depression with Structurally Differentiated Inhibitors

Authors and Affiliation:

Hyeijung Yoo[1], Kyung-Jun Boo[3], Lan Phuong Nguyen[2], Jong-Ik Hwang[2], Cheol Soon Lee[2], Soo Hyun Yang[1], Se Jin Jeon[4*], Hong-Rae Kim[2*] and Hyun Kim[1*]

[1]*Department of Anatomy, College of Medicine, Korea University, Seoul, 02841, Republic of Korea*

[2]*Department of Biomedical Sciences, College of Medicine, Korea University, Seoul, 02841, Republic of Korea*

[3]*Department of Pharmacology, College of Medicine and Center for Neuroscience Research, IBST, Konkuk University, Seoul, 05029, Republic of Korea*

[4]*Department of Pharmacology, College of Medicine, Hallym University, Chuncheon, Gangwon, 24252, Republic of Korea*

***Corresponding authors:**

Hyun Kim, MD. Ph.D.

Tel: 82-2-2286-1153

Fax: 82-2-929-5696

E-mail: kimhyun@korea.ac.kr

Hong-Rae Kim, Ph.D.

Tel: 82-2-3407-2097

E-mail: hrkim07@korea.ac.kr

Se Jin Jeon, Ph.D.

Tel: 82-33-248-2614

E-mail: sjinjeon@hallym.ac.kr

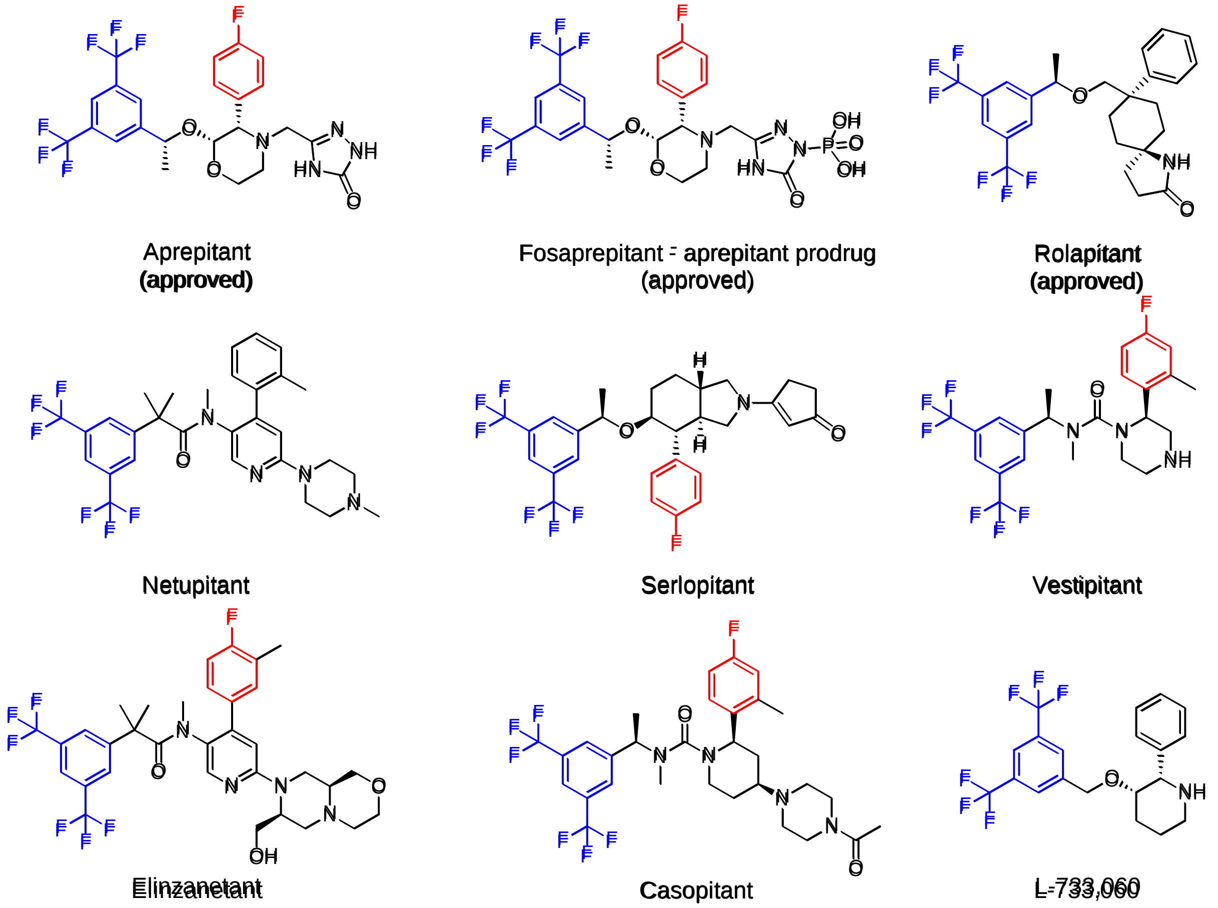

Supplementary Fig. 1 NK1R antagonists investigated in clinical trials. The characteristic 3,5-trifluoromethylphenyl (TFMP) structure is colored in blue, and the *para*-fluorophenyl group, another commonly observed motif, is shown in red.

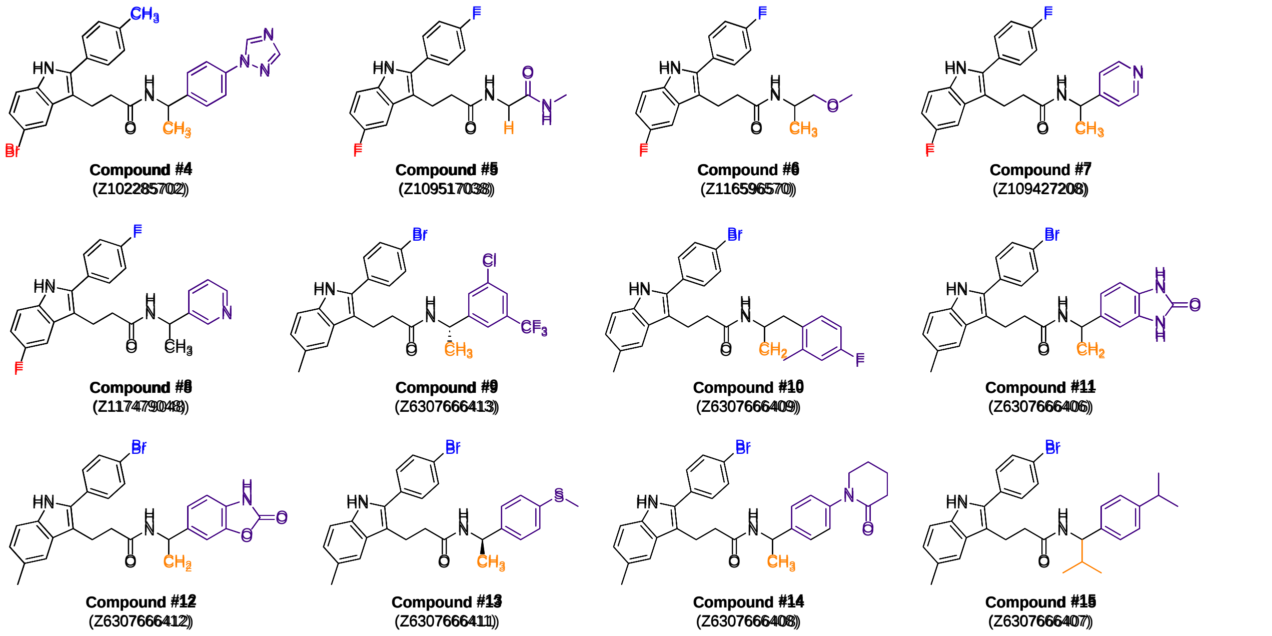

Supplementary Fig. 2. *in vitro* validated compounds from searching structural analogues of compound #1. Variations in structure are shown in different colors according to their positions.

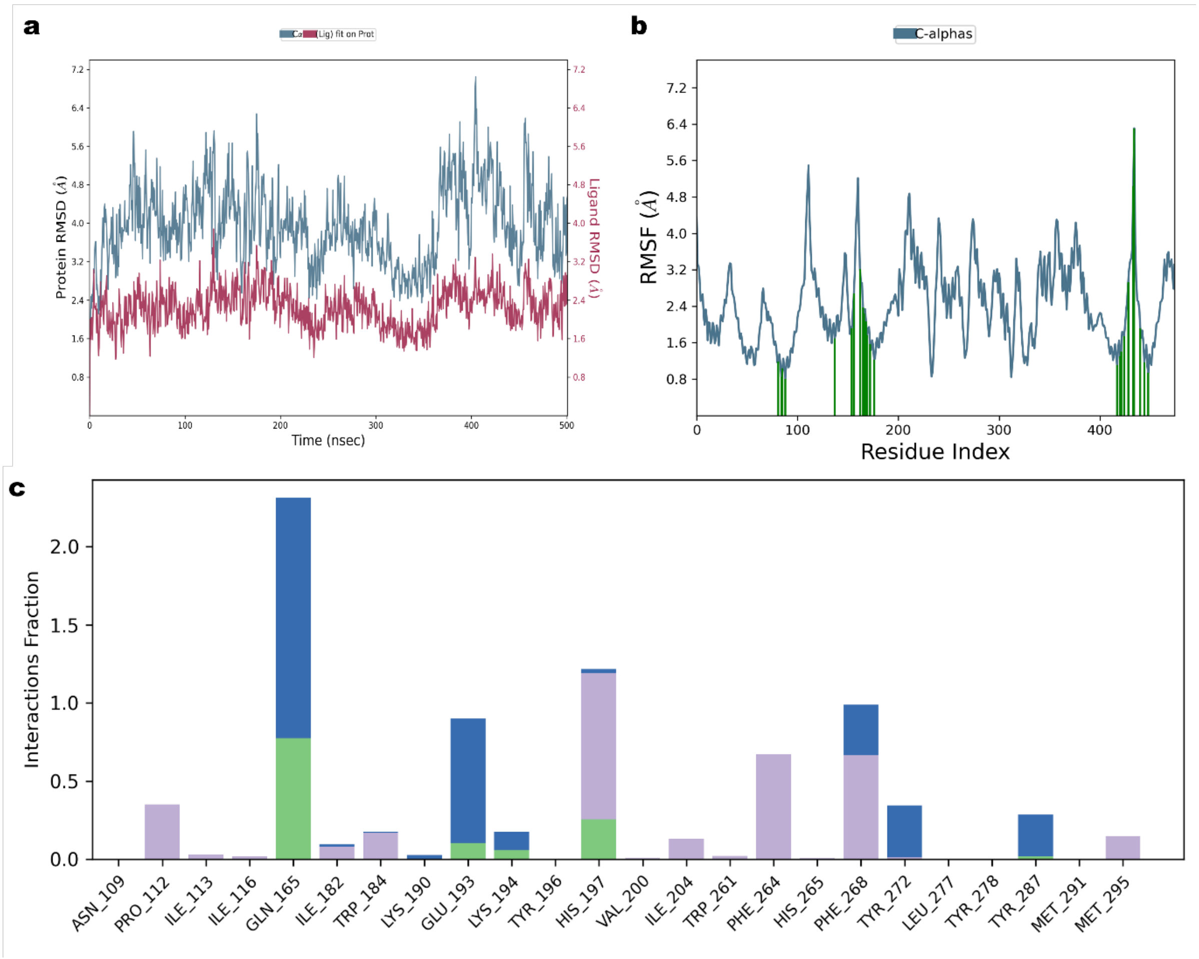

Supplementary Fig. 3. 500 ns molecular dynamics simulation of aprepitant. a Protein-ligand RMSD. Deviation of protein respect to frame 0 is indicated in blue and the ligand in red. **b** Fluctuation of each residue over the time course. The green lines indicate protein-ligand contacts. **c** Frequency of protein-ligand interactions. Hydrogen bonds are indicated in green, hydrophobic interaction in purple, water bridges in blue.

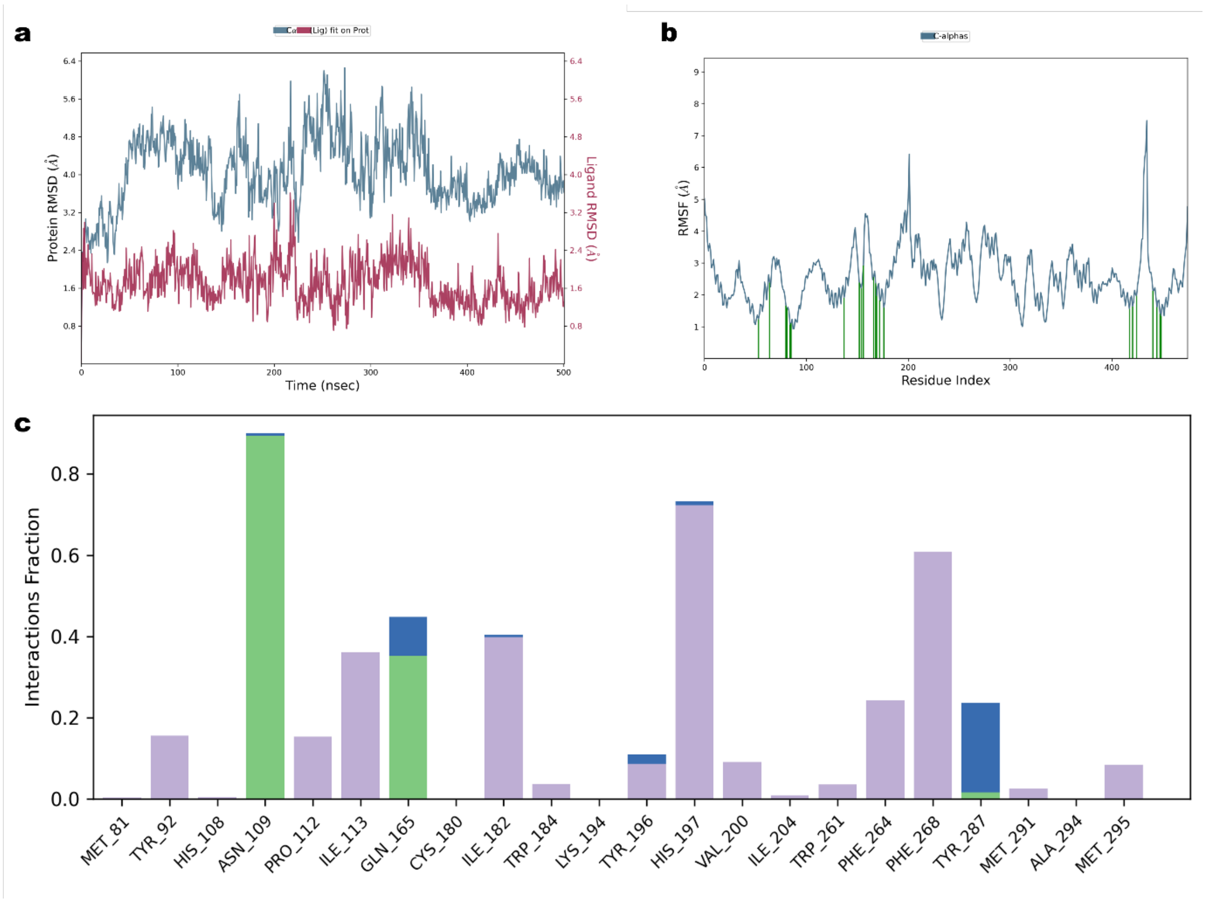

Supplementary Fig. 4. 500 ns molecular dynamics simulation of compound #15. a Protein-ligand RMSD. Deviation of protein respect to frame 0 is indicated in blue and the ligand in red. **b** Fluctuation of each residue over the time course. The green lines indicate protein-ligand contacts. **c** Frequency of protein-ligand interactions. Hydrogen bonds are indicated in green, hydrophobic interaction in purple, water bridges in blue.

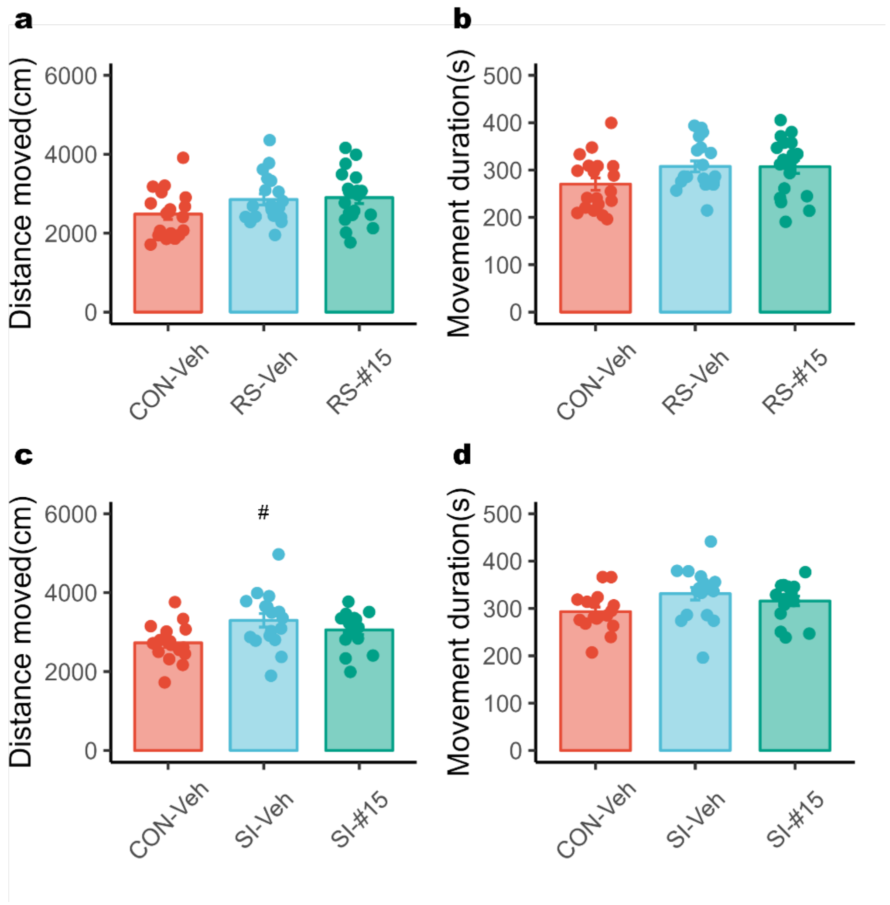

Supplementary Fig. 5. Open field test (OFT) results of RS and SI model The following groups were included: **a, b** CON (n = 19), RS (n = 19), RS + compound #15 (n = 19). **c, d** CON (n = 17), SI (n = 17), SI + compound #15 (n = 16). #$p < 0.05$ indicates statistical significance compared to vehicle-CON group.

ML method	Training		Test	
	R^2	RMSE	R^2	RMSE
Random forest	0.917	0.392	0.640	0.795
AutoQSAR	0.746	0.681	0.662	0.785
Deepchem	0.873	0.481	0.674	0.771
Simple regression	0.975	0.214	0.396	1.049

Supplementary Table 1. Machine-learning methods and their performance based on R^2 and RMSE

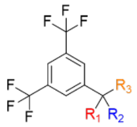

Name	Substituents		
	R_1	R_2	R_3
**L-733,060**	H	H	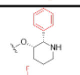
**Aprepitant**	H	(*R*)-Me	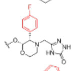
**Rolapitant**	H	(*R*)-Me	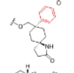
**Serlopitant**	H	(*R*)-Me	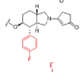
**Vestipitant**	H	(*R*)-Me	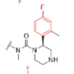
**Casopitant**	H	(*R*)-Me	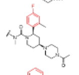
**Netupitant**	Me	Me	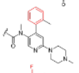
**Elinzanetant**	Me	Me	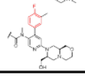

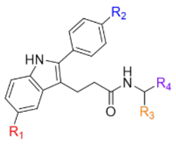

No.	Substituents				Calcium-6
	R_1	R_2	R_3	R_4	IC_{50} (μM)
**1**	F	OMe	Me	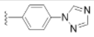	4.01
**4**	F	F	Me	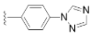	4.15
**5**	F	F	Me	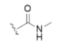	>50
**6**	F	F	Me	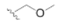	>50
**7**	F	F	Me	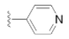	>50
**8**	Me	Br	Me	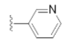	>50
**9**	Me	Br	(*S*)-Me	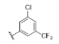	>50
**10**	Me	Br	Me	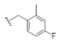	>50
**11**	Me	Br	Me	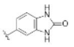	>50
**12**	Me	Br	Me	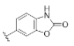	9.00
**13**	Me	Br	(*R*)-Me	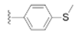	0.981
**14**	Me	Br	Me	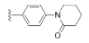	0.155
**15**	Me	Br	iPr	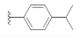	0.0795

Supplementary Table 2. Structural comparison of legacy NK1R antagonists and identified aryl indole analogues. Representative legacy NK1R antagonists containing the 3,5-bis-trifluoromethylphenyl (TFMP) group, including aprepitant, are shown on the left. The

aryl indole analogues identified in this study, which lack the TFMP group and do not share core scaffold similarity with legacy compounds, are shown on the right. This table illustrates the structural differentiation of the newly proposed compounds relative to existing NK1R antagonist classes.

Gene	Primer	Sequence	Size
GAPDH	FW	5'- ACC CAG AAG ACT GTG GAT GG -3'	171bp
	RV	5'- CAC ATT GGG GGT AGG AAC AC -3'	
IL-1β	FW	5'- CTG TGT CTT TCC CGT GGA CC -3'	200bp
	RV	5'- CAG CTC ATA TGG GTC CGA CA -3'	
TNF-α	FW	5'- CTG TAG CCC ACG TCG TAG CA -3'	198bp
	RV	5'- TGT GGG TGA GGA GCA CGT AG -3'	
IL-6	FW	5'- GAG GAT ACC ACT CCC AAC AGA CC -3'	141bp
	RV	5'- AAG TGC ATC ATC GTT GTT CAT ACA -3'	

Supplementary Table 3. Primer sets used for qRT-PCR.

	MW (g/mol)	Dipole	Volume (A3)	HBD	HBA	QLogPo/w	QlogBB	QPPCaco (nm/s)	QPPMDCK (nm/s)	QPlogKhsa	PSA	Rotatable Bonds
Criteria*	<450	1 – 12.5	500 - 2000	0	<5	4	-3 – 1.2	> 500	> 500	-1.5 – 1.5	70 – 90	< 6
Aprepitant	534	3.809	1361.255	2	8.4	3.738	-0.221	95.828	859.384	0.455	93.046	5
Compound #15	532	6.127	1644.983	2	2.5	8.139	-0.201	2463.655	5546.382	1.905	45.832	7

Supplementary Table 4. Predicted blood-brain barrier penetration capability of aprepitant and compound #15 [43]. Red color indicates values outside and green indicates values within the ideal range.
